# Characterization of a novel mutation in the *CRYBB2* gene associated with autosomal dominant congenital posterior subcapsular cataract in a Chinese family

**Published:** 2011-01-13

**Authors:** Ke Yao, Jinyu Li, Chongfei Jin, Wei Wang, Yanan Zhu, Xingchao Shentu, Qiwei Wang

**Affiliations:** Eye Center of the 2nd Affiliated Hospital, Medical College of Zhejiang University, Hangzhou, China

## Abstract

**Purpose:**

To identify the underlying genetic defect in four generations of a Chinese family affected with bilateral congenital posterior subcapsular cataracts.

**Methods:**

Clinical data from patients in the family were recorded by slit-lamp photography. Genomic DNA samples were extracted from peripheral blood of the pedigree members. Mutation screening was performed in the candidate gene by bidirectional sequencing of the amplified products. The mutation was verified by restriction fragment length polymorphism (RFLP) analysis.

**Results:**

The congenital cataract phenotype of the pedigree was identified as posterior subcapsular by slit-lamp photography. Sequencing of the candidate genes detected a heterozygous c.5C→T change in the coding region of the βB2-crystallin gene (*CRYBB2*), resulting in the substitution of a highly conserved alanine to valine (p. A2V). All nine family members affected with cataracts were positive for this change, but it was not observed in any of the unaffected members of the family. The transition resulted in the loss of a HaeIII restriction site in the affected members of the pedigree, which was present in the unaffected family members and in all of the 100 unrelated individuals tested.

**Conclusions:**

This study has identified a novel *CRYBB2* gene mutation, resulting in the amino substitution p. A2V in a Chinese family with posterior subcapsular congenital cataracts. This mutation is probably the causative lesion for the observed phenotype in this family.

## Introduction

Congenital cataracts, the loss of eye lens transparency, are a leading cause of visual impairment or blindness in childhood. Depending on regional socioeconomic development, its prevalence varies from 1−6 cases per 10,000 live births in industrialized countries [1,2] to 5−15 per 10,000 in the poorest areas of the world [3,4]. Globally, congenital cataracts account for approximately one-tenth of childhood blindness due to different causes including infections during embryogenesis, metabolic disorders (galactosemia), and gene defects [5]. As inherited cataracts correspond to 8−25% of congenital cataracts [6], genetic mutation is likely the most common cause. Although autosomal recessive and X-linked forms of inheritance also exist, autosomal dominance is the major form of inheritance of congenital cataracts [7]. Autosomal dominant congenital cataracts (ADCC) have been reported to be caused by mutations in 24 different genes to date: approximately half of the mutations are in the crystallin genes and a quarter in connexins genes, with the remainder divided among the genes for heat shock transcription factor-4 (*HSF4*), aquaporin-0 (*AQP0, MIP*), paired-like homeodomain 3 (*PITX3*), v-maf musculoaponeurotic fibrosarcoma oncogene homolog (*MAF*), chromatin modifying protein (*CHMP4B*), lens intrinsic membrane protein 2 (*LIM2*), beaded filament structural protein-2 (*BFSP2*) [5,8], the forkhead box protein E3 (*FOXE3*) [9], Drosophila eyes absent gene 1 (*EYA1*) [10], intermediate filament protein vimentin (*VIM*) [11], and carboxylic acid transporter family SLC16A12 [12].

According to the location of the lens opacities, and a detailed description of the shape and appearance, a comprehensive approach is to classify the complex spectrum of morphological variations of congenital cataracts. The cataract phenotype can be divided into the following categories: total, nuclear, cortical, anterior subcapsular, posterior subcapsular, lamellar, cerulean, pulverulent, sutural, coralliform, wedge-shaped, and polymorphic cataracts [5,13]. Mutations in different genes may be associated with similar phenotypes due to genetic heterogeneity [14]. To date, congenital posterior subcapsular cataracts have been related to these genes: *CRYAB* [15,16], *CRYBA1* [17], *GJA8*, receptor tyrosine kinase gene (*EPHA2*) [18], *PITX3* [19], and *CHMP4B* [20].

In this paper, a four-generation family affected with congenital posterior subcapsular cataracts was investigated in an attempt to identify the genetic defect associated with their cataract phenotype.

## Methods

### Clinical evaluation and DNA specimens

**Figure 1:**
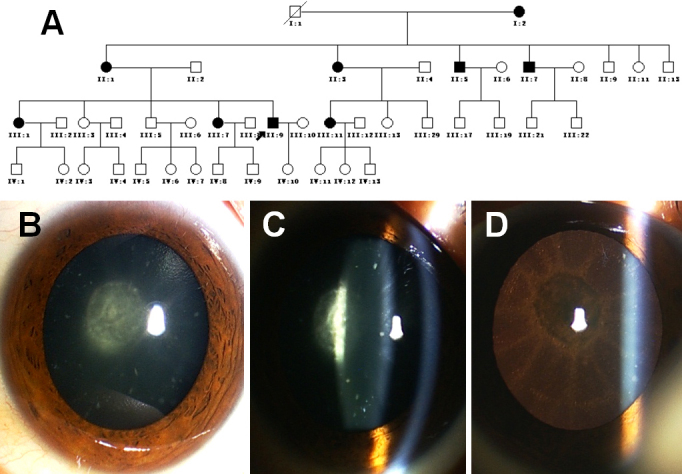
Clinical features of the family. **A**: Pedigree of the autosomal dominant congenital cataract. The proband is marked with an arrow. Squares and circles indicate males and females respectively. Black and white symbols represent affected and unaffected individuals, respectively. **B**, **C**, and **D**: Slit-lamp photograph of the proband (III:9) shows a posterior subcapsular cataract.

The four generation of the family suffering with ADCC were recruited from the Eye Center of Affiliated Second Hospital, College of Medicine, Zhejiang University, Hangzhou, China. Informed consent in accordance with the Zhejiang Institutional Review Board was obtained from all participants and the study protocol adhered to the tenets of the Declaration of Helsinki. In total, 27 individuals participated: nine affected and 18 unaffected. Of the 18, nine were spouses (Figure 1A). Also, 100 unrelated control subjects were recruited. Detailed medical histories were obtained by interviewing all individuals. All participants underwent ophthalmologic examinations, including visual acuity and slit-lamp examination with dilated pupils. Five of the affected members had undergone cataract extraction surgery. Phenotypes were documented by slit-lamp photography (Figure 1B-D). Blood samples were obtained by venipuncture, collected in Vacutainer tubes (Becton-Dickinson, Franklin Lakes, NJ) containing EDTA. Leukocyte genomic DNA was extracted using the QIAmp Blood kit (Qiagen, Duesseldorf, Germany).

### Mutation analysis

Genomic DNA samples from affected and unaffected members of the family were screened for mutations in *CRYAA*, *CRYAB*, *CRYBA1*, *CRYBB2, CRYGC, CRYGD, GJA3, GJA8, PITX3*, and *CHMP4B* by a combination of direct sequencing and RFLP analysis. The coding regions of candidate genes were amplified by polymerase chain reaction (PCR) using previously published primer sequences (Table 1) [9,20-26]. The cycling conditions for PCR were 95 °C preactivation for 5 min, 10 cycles of touchdown PCR with 0.5 °C down per cycle from 60 °C to 55 °C, followed by 30 cycles with denaturation at 95 °C for 25 s, annealing at 55 °C for 25 s, and extension at 72 °C for 40 s. PCR products were isolated by electrophoresis on 3% agarose gels and sequenced using the BigDye Terminator Cycle sequencing kit V 3.1 (ABI Applied Biosystems; Sangon Co, China) on an ABI PRISM 3730 Sequence Analyzer (ABI), according to the manufacturer’s instructions. Sequencing results were analyzed using Chromas 1.62 and compared with sequences from NCBI GenBank (*CRYAA*: 21q22.3; NM_000394, *CRYAB*: 11q22; NG_009824, *CRYBA1*: 17q11-q12; NM_005208, *CRYBB2*: 22q11.2; NM_000496, *CRYGC*: 2q33-q35; NM_020989, *CRYGD*: 2q33-q35; NM_006891.3, *GJA3:* 13q11-q13; NM_021954, *GJA8:* 1q21-q25; NM_005267, *PITX3*: 10q25; NM_005029, and *CHMP4B*: 20q11.22; NM_176812).

**Table 1:** Polymerase chain reaction primers and product sizes of human *CRYBB2* genes.

**Name**	**Primer sequence (5′-3′)**	**Tm (°C)**	**Product size (bp)**
CRYBB22F	5′-CCAGGTCCTCACTGCTGCTTC-3′	60.34	205
CRYBB22R	5′-CCCATTTTACAGAAGGGCAAC-3′	58.01	
CRYBB23F	5′-ACCCTTCAGCATCCTTTGG-3′	57.56	314
CRYBB23R	5′-GCAGACAGGAGCAAGGGTAG-3′	61.9	
CRYBB24F	5′-GCTTGGAGTGGAACTGACCTG-3′	61.92	244
CRYBB24R	5′-GGCAGAGAGAAAGTAGGATGATG-3′	61.98	
CRYBB25F	5′-GCCCCCTCACCCATACTC-3′	61.86	242
CRYBB25R	5′-CCCCAGAGTCTCAGTTTCCTG-3′	61.92	
CRYBB26F	5′CCTAGTGGCTTATGGATGCTC-3′	59.97	347
CRYBB26R	5′-TCTTCACTTGGAGGTCTGGAG-3′	59.97	

### Restriction fragment length polymorphism analysis

Genetic variations were confirmed by the absence of cleavage sites for the restriction enzyme HaeIII in affected family members. For controls, we analyzed 100 DNA samples from ophthalmologically normal individuals of the same ethnic background as the family members. PCR products of exon 2 of the CRYBB2 gene were digested for 10 h at 37 °C with HaeIII (TAKARA, Dalian, China), then electrophoresized in 5% polyacrylamide gels and analyzed under ultraviolet (UV) light.

### Computational algorithms

Computational methods have been shown to be effective in predicting whether a specific amino acid substitution of a protein sequence is deleterious or neutral to the function of the protein. Three methods were used: Sorting Intolerant from Tolerant amino acid substitutions (SIFT), Polymorphism Phenotyping (PolyPhen), and Grantham score difference (Align-GVGD).

SIFT uses sequence homology to predict whether an amino acid substitution will affect protein function and, thus, potentially contribute to a disease. It is based on the approach that important amino acids tend to be highly conserved across species. SIFT, which assigns scores from 0 to 1, predicts substitutions with scores less than 0.05 as deleterious, whereas those greater than or equal to 0.05 are considered to be tolerated [27]. PolyPhen takes into account the evolutionary conservation of the amino acid subjected to the mutation and the physicochemical characteristics of the wild-type and mutated amino acid residue and the consequence of the amino acid change for the structural properties of the protein [6]. Grantham Variation (GV) measures the degree of biochemical variation among amino acids found at a given position in the multiple sequence alignment: Grantham Deviation (GD) reflects the 'biochemical distance' of the mutant amino acid from the observed amino acid at a particular position (given by GV). Align-GVGD can be used to predict the transactivation activity of each missense substitution [28]. A value of GV=0 corresponds to a residue that is invariant in the alignment, a value of GV of 60–65 is the upper limit of conservative variation across species, and a value of GV>100 is indicative of positions that are under little functional constraint. A value of GD=0 corresponds to a missense substitution that is within the cross-species range of variation at its position in the protein; at invariant positions (GV=0); GD=60–65 is the upper limit of a conservative missense substitution [29]. Additionally, for hydropathy analysis we used Kyte-Doolittle hydropathy plots. All hydropathies for both wild-type and mutants were calculated in a default window size of 7.

## Results

### Clinical evaluation

The cataract exhibited an autosomal dominant inheritance pattern in the family under investigation (Figure 1A). Opacification of the lens was bilateral in all affected individuals. There was no family history of other ocular or systemic abnormalities. Five of the nine patients had undergone lens surgery. Slit-lamp examination of the eyes in the remaining patients without cataract removal showed opacity in the posterior subcapsular region with additional dot-like opacification in its cortex of bilateral lenses (Figure 1B-D). Visual acuity in the unoperated eyes of those affected individuals ranged from 0.6 to 0.02. Most affected individuals noticed their visual impairments in their twenties, and then their visual acuity decreased gradually until surgery was required to improve their visual function after the age of 40.

### Mutation screening

**Figure 2:**
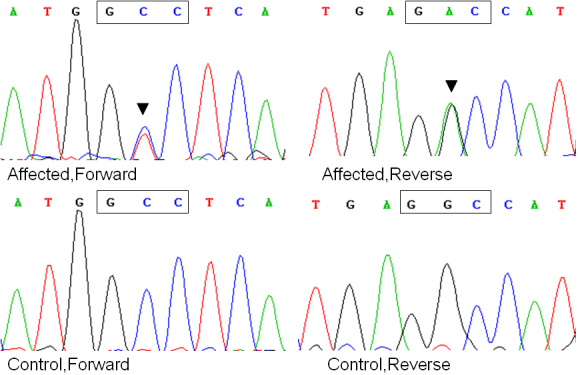
Forward and reverse sequence analysis of the affected and unaffected individuals in the ADCC Chinese family, showing a heterozygous mutation (c.5C>T) in exon 2 of *CRYBB2* (black triangles).

Bidirectional sequencing of the coding regions of the candidate genes showed a heterozygous change, C>T (Figure 2), at position 5 (c.5C>T) of the *CRYBB2* gene in all nine affected individuals, leading to the replacement of a highly conserved alanine with valine at the second amino acid position (p. A2V). This substitution was not seen in the unaffected individuals or in the 100 unrelated control subjects (200 chromosomes) from the same Chinese population (data not shown).

### Restriction fragment length polymorphism analysis

**Figure 3:**
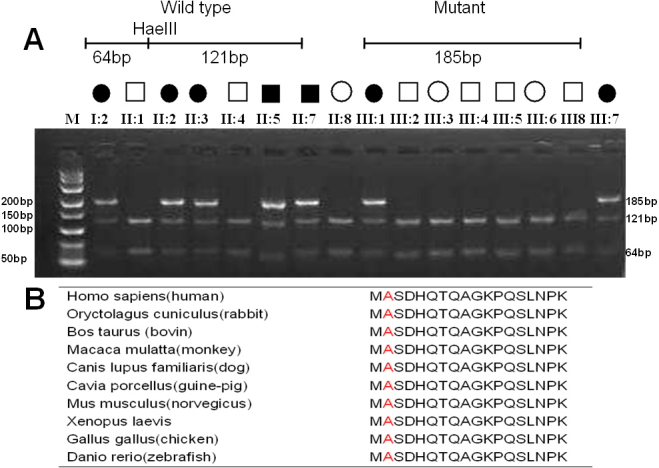
The *CRYBB2* mutation cosegregates with the disease in the family. **A**: Restriction fragment length analysis (RFLP) showing the loss of the HaeIII restriction site in heterozygous individuals with the A2V mutation (185 bp) but it was present in unaffected individuals (121 bp and 64 bp). **B**: Multiple-sequence alignment in CRYBB2 from different species reveals that codon 2, where the mutation (p. A2V) occurred, is highly conserved (highlighted in red).

The mutation was confirmed by a HaeIII digest of the PCR-amplified exon 2 of the *CRYBB2* gene. This mutation resulted in the loss a HaeIII restriction site in all the affected members of the Chinese family under investigation, but was not detected in the 100 unrelated normal controls or unaffected pedigree members (Figure 3A).

### Multiple-sequence alignment and mutation analysis

Using the NCBI websites, a multiple sequence alignment showed that the alanine at position 2 of human the CRYBB2 protein (*Homo sapiens*, NP_000487.1) is highly conserved in various species including *Oryctolagus cuniculus* (NP_001082786.1), *Bos taurus* (NP_777232.1), *Macaca mulatta* (NP_001116366.1), *Canis lupus familiaris* (NP_001041578.1), *Cavia porcellus* (NP_001166542.1), *Mus musculus* (NP_031799.1), *Xenopus laevis* (NP_001086491.1), *Gallus gallus* (NP_990506.2), and *Danio rerio* (NP_001018138.1; Figure 3B).

### Computational analysis

**Figure 4:**
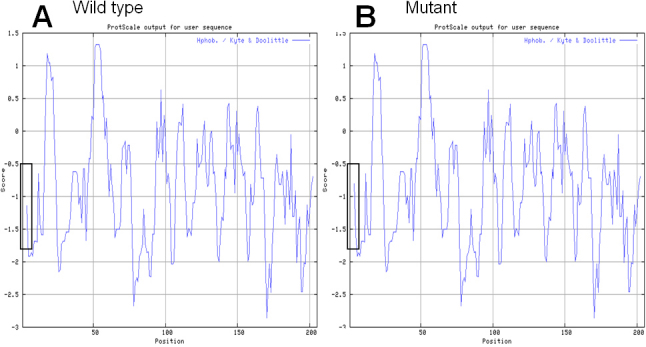
Hydropathy analysis of  the mutant protein. Kyte-Doolittle hydropathy plot of CRYBB2WT (**A**) and CRYBB2A2V (**B**). The x-axis represents position of amino acids. The y-axis represents the hydropathy value in a default window size of 7. The region of interest is marked by white boxes. The increase in hydrophobicity in the mutant form is evident.

Computational protein analysis of A2V CRYBB2 revealed the following results: the SIFT method revealed a score of 0.00, meaning the substitution is intolerant. PolyPhen analysis produced a score of 1.603, which is predicted to be “probably damaging.” Finally, Align-GVGD showed a score of GV0.00, GD65.28, which belongs to class C65 and means “most likely to interfere with function.” All of these results indicated the A2V substitution was likely deleterious and possibly contributed to the disease. The Kyte-Doolittle algorithm for hydrophobicity analysis showed the local hydrophobicity at and near the altered amino acid was increased (Figure 4).

## Discussion

Crystallins are known to constitute about 90% of the water-soluble proteins of the lens and contribute to transparency and refractive properties, due to a uniform concentration gradient in the lens. The vertebrate crystallins are divided into two families: α-crystallins and the β- and γ-crystallin families [30,31]. The β- and γ-crystallins share a common feature of anti-parallel β sheets in the proteins, referred to as the “Greek key motif.” The Greek key motif are agreed to be among the most stable of structures in proteins. Computer-based analyses suggest that they form an interdomain association: intramolecular in the γ-crystallins, and intermolecular in the β-crystallins. The detailed analysis of the mutations in the β- and γ-crystallin encoding genes might help to identify those amino acids which are the important corner stones for this function [32]. A second functional aspect of the Greek key motif is its Ca^2+^ binding properties. A human lens model of cortical cataract had been developed by Sanderson and his coworkers to study the role of Ca^2+^ in cataractogenesis [33].Recently, Fischer et al. [34] reported on the effects of β- and γ-crystallins in axon regeneration of retinal ganglion cells.

The β-crystallin gene consists of six exons; the first exon is not translated, the second exon encodes the NH_2_-terminal extension, and the subsequent four exons are responsible for one Greek key motif each [35]. Mutations in the *CRYBB2* gene in humans and mice have been reported to induce genetic cataracts [8,23,36-49] (Table 2). Previously, nine geographically distinct families have been reported with the same nonsense mutation (Q155X) in exon 6 of the *CRYBB2* gene, which is associated with diverse phenotypes. This mutation creates a premature stop codon (Q155X) and results in an in-frame stop codon at nucleotide 475 of exon 6 that may cause a truncation of 50 amino acids from the COOH-terminus of βB2-crystallin, which destabilizes the domain structure of betaB2-crystallin. Using a mammalian two-hybrid system assay, spectroscopy (circular dichroism and fluorescence) and fast performance liquid chromatography (FPLC), the Q155X mutant shows not only decreased ordered structure and stability, but also a decrease in protein–protein interactions with βB2-crystallin mutant, which might contribute to cataract formation [50]. This mutation is explained by a gene conversion mechanism between the *CRYBB2* gene and its pseudogene *CRYBB2P1*. The diversity of the phenotypes may be caused by variations in the promoter region, possibly influencing the expression of the CRYBB2 protein in the lens or other crystallin genes as modifiers from surrounding loci [37,39]. The other mutations were found in exons 3, 5, and 6. A2V in the *CRYBB2* gene characterized here is the first reported mutation in exon 2 (NH_2_-terminal extension) of the *CRYBB2* gene.

**Table 2:** Previous *CRYBB2* gene mutations associated with congenital cataracts.

**Bp exchange**	**Aa exchange**	**Biologic consequence**	**Origin of family**	**Reference**
**Human mutations**				
c. G607A	p. V187M	Nuclear cataract	Basotho	[36]
c. C475T	p. Q155X	Sutural opacity and fish tail-like branches	American	[37]
c. C475T	p. Q155X	Cerulean	American	[8]
c. C475T	p. Q155X	ADCC	Canadian	[38]
c. C475T	p. Q155X	ADCC	Chilean	[39]
c. C475T	p. Q155X	Progressive polymorphic coronary ADCC	Indian	[40]
c. C92T	p. S31W	Coronary cataract	Chinese	[41]
c. C475T	p. Q155X	Cerulean ADCC	Chinese	[42]
c. C475T	p. Q155X	Progressive polymorphic	Chinese	[43]
c. G465T	p. W151C	Central nuclear	Indian	[44]
c. A383T	p. D128V	Dominant ring-shaped cortical cataract	Germany	[45]
c.C489A	p. Y159X	ADCC	Danish	[46]
c.C433T	p. R145W	ADCC	Danish	[46]
c.A440G	p. Q147R	ADCC	Danish	[46]
c.C449T	p. T150M	ADCC	Danish	[46]
**Mouse mutations**				
Intron 5:_57 A->T	Splicing: 19 new amino acids in front of exon 6	Progressive cataract		[47]
c. T560A	p. V187Q	Progressive cataract		[35]
585–587 Deletion	195–198 ΔQSVR	Progressive cataract		[48,49]

βB2-crystallin, the major component of β-crystallin, is a homodimer at low concentrations, and can form a heterodimer with other beta-crystallins under physiologic conditions [51]. Based on the structure from X-ray refraction studies for homodimer βB2-crystallin, each subunit includes 16 β-strands, eight in the NH_2_-terminal domain and eight in the COOH-terminal domain [51]. The results of NMR spectroscopic studies indicate that the terminal extensions of beta B2-crystallin appear to be of little ordered conformation, are accessible to solvent and flex freely from the main body of the protein [52].Earlier studies indicated that the NH_2_-terminal extension of β-crystallin played an important role in oligomerization [53].Recent experimental data suggest that the NH_2_- and COOH-terminal arms appear to be involved in preventing the formation of higher homo- and hetero oligomers [54].The long and flexible NH_2_-terminal extension of the *CRYBB2* (PDB structure 1YTQ) might be critical for mediating protein interactions. Thus, the A2V substitution may influence homo- and heteromolecular interactions, which would contribute to cataract formation. In addition, lens crystallins are known to be susceptible to a wide variety of post-translational modifications such as acetylation, deamidation, methylation, oxidation, phosphorylation, and truncation of terminal extensions by thiol proteases. Age-related proteolytic processing of human lens β-crystallins occurs mainly at the NH_2_-terminal extensions [55]. This single base substitution may play a role in post-translational modifications of crystallins, which would lead to protein structure and function alteration.

By the SIFT, PolyPhen, and Align-GVGD programs, we evaluated the possible effect of this amino acid substitution (p. A2V) on βB2-crystallin protein function. The programs were used to determine whether a specific amino acid substitution would lead to an altered protein structure and function, based on sequence homology and structural information. As the isolated predictive value of these programs can be increased by their combination [6,56], it is believed that the A2V mutation alters the protein structure of the βB2-crystallin protein to such an extent it may contribute to the disease.

Also, hydropathy analysis revealed a variation in the physicochemical properties of the critical region in the A2V mutant (Figure 4B) compared with wild-type βB2-crystallin (Figure 4A). The environment surrounding the amino acid “V” in the mutant protein is more hydrophobic. Thus, the increase in hydrophobicity in the mutant form might affect the solubility of the mutant protein and hence contribute to cataract formation.

### Conclusions

A novel A2V mutation of the CRYBB2 protein was identified and characterized in a Chinese family presenting with the posterior subcapsular type of congenital cataract. Further experiments on this cataract-related genetic defect and the factors that modify their variable phenotypes will improve our understanding of the mechanism of cataract formation and illuminate the developmental biology and biochemistry of the lens.

## References

[1] Rahi JS Sripathi S Gilbert CE Foster A Childhood blindness in India: causes in 1318 blind school students in nine states. Eye (Lond) 1995 9 545 50 8543070 10.1038/eye.1995.137

[2] Gilbert C Foster A Childhood blindness in the context of VISION 2020–the right to sight. Bull World Health Organ 2001 79 227 32 11285667 PMC2566382

[3] Apple DJ Ram J Foster A Peng Q Elimination of cataract blindness: a global perspective entering the new millenium. Surv Ophthalmol 2000 45 S1 196 11291895

[4] Francis PJ Berry V Bhattacharya SS Moore AT The genetics of childhood cataract. J Med Genet 2000 37 481 8 10882749 10.1136/jmg.37.7.481 PMC1734631

[5] Reddy MA Francis PJ Berry V Bhattacharya SS Moore AT Molecular genetic basis of inherited cataract and associated phenotypes. Surv Ophthalmol 2004 49 300 15 15110667 10.1016/j.survophthal.2004.02.013

[6] Santana A Waiswol M Arcieri ES Cabral de Vasconcellos JP Barbosa de Melo M Mutation analysis of CRYAA, CRYGC, and CRYGD associated with autosomal dominant congenital cataract in Brazilian families. Mol Vis 2009 15 793 800 19390652 PMC2671581

[7] Hejtmancik JF Congenital cataracts and their molecular genetics. Semin Cell Dev Biol 2008 19 134 49 18035564 10.1016/j.semcdb.2007.10.003 PMC2288487

[8] Devi RR Yao W Vijayalakshmi P Sergeev YV Sundaresan P Hejtmancik JF Crystallin gene mutations in Indian families with inherited pediatric cataract. Mol Vis 2008 14 1157 70 18587492 PMC2435160

[9] Brémond-Gignac D Bitoun P Reis LM Copin H Murray JC Semina EV Identification of dominant FOXE3 and PAX6 mutations in patients with congenital cataract and aniridia. Mol Vis 2010 16 1705 11 20806047 PMC2927439

[10] Azuma N Hirakiyama A Inoue T Asaka A Yamada M Mutations of a human homologue of the Drosophila eyes absent gene (EYA1) detected in patients with congenital cataracts and ocular anterior segment anomalies. Hum Mol Genet 2000 9 363 6 10655545 10.1093/hmg/9.3.363

[11] Müller M Bhattacharya SS Moore T Prescott Q Wedig T Herrmann H Magin T Dominant cataract formation in association with a vimentin assembly disrupting mutation. Hum Mol Genet 2009 18 1052 7 19126778 10.1093/hmg/ddn440

[12] Kloeckener-Gruissem B Vandekerckhove K Nurnberg G Neidhardt J Zeitz C Nurnberg P Schipper I Berger W Mutation of solute carrier SLC16A12 associates with a syndrome combining juvenile cataract with microcornea and renal glucosuria. Am J Hum Genet 2008 82 772 9 18304496 10.1016/j.ajhg.2007.12.013 PMC2427214

[13] Amaya L Taylor D Russell-Eggitt I Nischal KK Lengyel D The morphology and natural history of childhood cataracts. Surv Ophthalmol 2003 48 125 44 12686301 10.1016/s0039-6257(02)00462-9

[14] Lorenz B Genetic examination in cases of congenital cataract. Ophthalmologe 2007 104 559 65 17571268 10.1007/s00347-007-1557-2

[15] Liu M Ke T Wang Z Yang Q Chang W Jiang F Tang Z Li H Ren X Wang X Wang T Li Q Yang J Liu J Wang QK Identification of a CRYAB mutation associated with autosomal dominant posterior polar cataract in a Chinese family. Invest Ophthalmol Vis Sci 2006 47 3461 6 16877416 10.1167/iovs.05-1438

[16] Veromann S Theoretical considerations regarding the study “Alpha-B crystallin gene (CRYAB) mutation causes dominant congenital posterior polar cataract in humans”. Am J Hum Genet 2002 71 684 5 12227334 10.1086/342207 PMC379206

[17] Gu Z Ji B Wan C He G Zhang J Zhang M Feng G He L Gao L A splice site mutation in CRYBA1/A3 causing autosomal dominant posterior polar cataract in a Chinese pedigree. Mol Vis 2010 16 154 60 20142846 PMC2817011

[18] Zhang T Hua R Xiao W Burdon KP Bhattacharya SS Craig JE Shang D Zhao X Mackey DA Moore AT Luo Y Zhang J Zhang X Mutations of the EPHA2 receptor tyrosine kinase gene cause autosomal dominant congenital cataract. Hum Mutat 2009 30 E603 11 19306328 10.1002/humu.20995

[19] Burdon KP McKay JD Wirth MG Russell-Eggit IM Bhatti S Ruddle JB Dimasi D Mackey DA Craig JE The PITX3 gene in posterior polar congenital cataract in Australia. Mol Vis 2006 12 367 71 16636655

[20] Shiels A Bennett TM Knopf HL Yamada K Yoshiura K Niikawa N Shim S Hanson PI CHMP4B, a novel gene for autosomal dominant cataracts linked to chromosome 20q. Am J Hum Genet 2007 81 596 606 17701905 10.1086/519980 PMC1950844

[21] Vanita V Singh JR Hejtmancik JF Nurnberg P Hennies HC Singh D Zhang X A novel fan-shaped cataract-microcornea syndrome caused by a mutation of CRYAA in an Indian family. Mol Vis 2006 12 518 22 16735993

[22] Lu S Zhao C Jiao H Kere J Tang X Zhao F Zhang X Two Chinese families with pulverulent congenital cataracts and Delta G91 CRYBA1 mutations. Mol Vis 2007 13 1154 60 17653060

[23] Litt M Carrero-Valenzuela R LaMorticella DM Schultz DW Mitchell TN Kramer P Maumenee IH Autosomal dominant cerulean cataract is associated with a chain termination mutation in the human beta-crystallin gene CRYBB2. Hum Mol Genet 1997 6 665 8 9158139 10.1093/hmg/6.5.665

[24] Zhang LY Yam GHF Fan DSP Tam POS Lam DSC Pang CP A novel deletion variant of gamma D-crystallin responsible for congenital nuclear cataract. Mol Vis 2007 13 2096 104 18079686

[25] Hansen L Yao WL Eiberg H Funding M Riise R Kjaer K Hejtmancik JF Rosenberg T The congenital “ant-egg” cataract phenotype is caused by a missense mutation in connexin46. Mol Vis 2006 12 1033 9 16971895

[26] Schmidt W Klopp N Illig T Graw J A novel GJA8 mutation causing a recessive triangular cataract. Mol Vis 2008 14 851 6 18483562 PMC2375854

[27] Ng PC Henikoff S SIFT: Predicting amino acid changes that affect protein function. Nucleic Acids Res 2003 31 3812 4 12824425 10.1093/nar/gkg509 PMC168916

[28] Mathe E Olivier M Kato S Ishioka C Hainaut P Tavtigian SV Computational approaches for predicting the biological effect of p53 missense mutations: a comparison of three sequence analysis based methods. Nucleic Acids Res 2006 34 1317 25 16522644 10.1093/nar/gkj518 PMC1390679

[29] Tavtigian SV Byrnes GB Goldgar DE Thomas A Classification of rare missense substitutions, using risk surfaces, with genetic- and molecular-epidemiology applications. Hum Mutat 2008 29 1342 54 18951461 10.1002/humu.20896 PMC3938023

[30] Graw J Cataract mutations and lens development. Prog Retin Eye Res 1999 18 235 67 9932285 10.1016/s1350-9462(98)00018-4

[31] Andley UP Crystallins in the eye: Function and pathology. Prog Retin Eye Res 2007 26 78 98 17166758 10.1016/j.preteyeres.2006.10.003

[32] Graw J Genetics of crystallins: cataract and beyond. Exp Eye Res 2009 88 173 89 19007775 10.1016/j.exer.2008.10.011

[33] Sanderson J Marcantonio JM Duncan G A human lens model of cortical cataract: Ca2+-induced protein loss, vimentin cleavage and opacification. Invest Ophthalmol Vis Sci 2000 41 2255 61 10892870

[34] Fischer D Hauk TG Muller A Thanos S Crystallins of the beta/gamma-superfamily mimic the effects of lens injury and promote axon regeneration. Mol Cell Neurosci 2008 37 471 9 18178099 10.1016/j.mcn.2007.11.002

[35] Graw J Loster J Soewarto D Fuchs H Reis A Wolf E Balling R Hrabé de Angelis M Aey2, a new mutation in the betaB2-crystallin-encoding gene of the mouse. Invest Ophthalmol Vis Sci 2001 42 1574 80 11381063

[36] Mothobi ME Guo SR Liu YY Chen Q Yussuf AS Zhu XL Fang Z Mutation analysis of congenital cataract in a Basotho family identified a new missense allele in CRYBB2. Mol Vis 2009 15 1470 5 19649175 PMC2718852

[37] Vanita SV Reis A, Jung M, Singh D, Sperling K, Singh JR, Bürger J. A unique form of autosomal dominant cataract explained by gene conversion between beta-crystallin B2 and its pseudogene. J Med Genet 2001 38 392 6 11424921 10.1136/jmg.38.6.392 PMC1734905

[38] Gill D Klose R Munier FL McFadden M Priston M Billingsley G Ducrey N Schorderet DF Héon E Genetic heterogeneity of the Coppock-like cataract: A mutation in CRYBB2 on chromosome 22q11.2. Invest Ophthalmol Vis Sci 2000 41 159 65 10634616

[39] Bateman JB von-Bischhoffshaunsen FRB Richter L Flodman P Burch D Spence MA Gene conversion mutation in crystallin, beta-B2 (CRYBB2) in a Chilean family with autosomal dominant cataract. Ophthalmology 2007 114 425 32 17234267 10.1016/j.ophtha.2006.09.013

[40] Li FF Zhu SQ Wang SZ Gao C Huang SZ Zhang M Ma X Nonsense mutation in the CRYBB2 gene causing autosomal dominant progressive polymorphic congenital coronary cataracts. Mol Vis 2008 14 750 5 18449377 PMC2335123

[41] Lou D Tong JP Zhang LY Chiang SW Lam DS Pang CP A novel mutation in CRYBB2 responsible for inherited coronary cataract. Eye (Lond) 2009 23 1213 20 18617901 10.1038/eye.2008.222

[42] Wang L Lin H Gu JZ Su H Huang SZ Qi Y Autosomal-Dominant Cerulean Cataract in a Chinese Family Associated with Gene Conversion Mutation in Beta-B2-Crystallin. Ophthalmic Res 2009 41 148 53 19321936 10.1159/000209668

[43] Yao K Tang X Shentu X Wang K Rao H Xia K Progressive polymorphic congenital cataract caused by a CRYBB2 mutation in a Chinese family. Mol Vis 2005 11 758 63 16179907

[44] Santhiya ST Manisastry SM Rawlley D Malathi R Anishetty S Gopinath PM Vijayalakshmi P Namperumalsamy P Adamski J Graw J Mutation analysis of congenital cataracts in Indian families: Identification of SNPs and a new causative allele in CRYBB2 gene. Invest Ophthalmol Vis Sci 2004 45 3599 607 15452067 10.1167/iovs.04-0207

[45] Pauli S Soker T Klopp N Illig T Engel W Graw J Mutation analysis in a German family identified a new cataract-causing allele in the CRYBB2 gene. Mol Vis 2007 13 962 7 17653036 PMC2774456

[46] Hansen L Mikkelsen A Nurnberg P Nurnberg G Anjum I Eiberg H Rosenberg T Comprehensive mutational screening in a cohort of Danish families with hereditary congenital cataract. Invest Ophthalmol Vis Sci 2009 50 3291 303 19182255 10.1167/iovs.08-3149

[47] Ganguly K Favor J Neuhauser-Klaus A Sandulache R Puk O Beckers J Horsch M Schädler S Vogt Weisenhorn D Wurst W Graw J Novel allele of crybb2 in the mouse and its expression in the brain. Invest Ophthalmol Vis Sci 2008 49 1533 41 18385073 10.1167/iovs.07-0788

[48] Duprey KM Robinson KM Wang Y Taube JR Duncan MK Subfertility in mice harboring a mutation in betaB2-crystallin. Mol Vis 2007 13 366 73 17392687 PMC2642919

[49] Chambers C Russell P Deletion mutation in an eye lens beta-crystallin. An animal model for inherited cataracts. J Biol Chem 1991 266 6742 6 1707874

[50] Liu BF Liang J Interaction and biophysical properties of human lens Q155*beta B2-crystallin mutant. Mol Vis 2005 11 321 7 15889016

[51] Liu BF Liang JJ Domain interaction sites of human lens betaB2-crystallin. J Biol Chem 2006 281 2624 30 16319073 10.1074/jbc.M509017200

[52] Carver JA Cooper PG Truscott RJ 1H-NMR spectroscopy of beta B2-crystallin from bovine eye lens. Conformation of the N- and C-terminal extensions. Eur J Biochem 1993 213 313 20 8477703 10.1111/j.1432-1033.1993.tb17764.x

[53] Berbers GA Hoekman WA Bloemendal H de Jong WW Kleinschmidt T Braunitzer G Proline- and alanine-rich N-terminal extension of the basic bovine beta-crystallin B1 chains. FEBS Lett 1983 161 225 9 6617875 10.1016/0014-5793(83)81013-8

[54] Trinkl S Glockshuber R Jaenicke R Dimerization of beta B2-crystallin: the role of the linker peptide and the N- and C-terminal extensions. Protein Sci 1994 3 1392 400 7833801 10.1002/pro.5560030905 PMC2142935

[55] Dolinska MB Sergeev YV Chan MP Palmer I Wingfield PT N-terminal extension of beta B1-crystallin: identification of a critical region that modulates protein interaction with beta A3-crystallin. Biochemistry 2009 48 9684 95 19746987 10.1021/bi9013984 PMC2764403

[56] Chan PA Duraisamy S Miller PJ Newell JA McBride C Bond JP Raevaara T Ollila S Nyström M Grimm AJ Christodoulou J Oetting WS Greenblatt MS Interpreting missense variants: comparing computational methods in human disease genes CDKN2A, MLH1, MSH2, MECP2, and tyrosinase (TYR). Hum Mutat 2007 28 683 93 17370310 10.1002/humu.20492

